# Supplementary material for: Continued selection on cryptic SARS-CoV-2 observed in Missouri wastewater
Source: PLoS Pathog. 2023 Dec 28;19(12):e1011688. doi: 10.1371/journal.ppat.1011688 (PMC10781090; doi:10.1371/journal.ppat.1011688)
Supplement: Attachment — Submitted filename: Response.docx [file ppat.1011688.s001.docx]

**Part I - Summary**
Please use this section to discuss strengths/weaknesses of study, novelty/significance, general execution and scholarship.

Reviewer #1: 
The main concern I have is whether this brief, observational study is appropriate for PLOS Pathogens. Although the work will no doubt be of interest to many people in the virology field, and beyond, the observation of convergent evolution in the Spike gene is no longer novel or original, but rather has become commonplace and is to-be-expected. Moreover, the manuscript does not include any studies of how these amino acid changes impact viral entry, antibody escape, et cetera. Most reports evaluating novel variants include functional, "wet lab" analyses, at least using pseudotyped viruses, to evaluate the phenotypic outcomes of the observed nucleotide changes. That is lacking here. Though again, the authors only have the RBD, so there'd be a question of what sort of Spike the authors should place these changes into for any proposed laboratory studies.

***We don’t disagree with any of the reviewer’s comments. We weren’t sure if PLoS Pathogens was the right journal, but we reached out to one of the PP editors and they encouraged us to submit here. There remains debate in the field about where Omicron came from there remains groups that believe it was likely engineered. While I don’t yield any credence in such beliefs, when I have data that demonstrates that something that looks like very much like Omicron can develop naturally, I like to share it.***

***In response for the request for wet lab experiments, we built the RBD of the MO45 cryptic lineage and generated pseudoparticles (Figure 3). This demonstrated both that the cryptic spike is functional, and that it became highly resistant to neutralizing antibodies that were able to neutralize its predecessor.***

Otherwise, the authors methodology and approach are straightforward, and the manuscript is well written. The authors' take-home point /argument that surveillance of cryptic lineages is valuable / worthwhile in providing a signal of changes yet-to-come in circulating variants is valid.

Reviewer #2: In this short report Gregory and coauthors describe the long-term and ongoing detection of a ‘cryptic’ wastewater lineage – what is presumed to be a undetected chronic infection, perhaps in an immunocompromised host. The authors draw parallels between mutations appearing in this lineage and the same sites of substitutions appearing at later dates in Omicron or Omicron sublineages. This topic is of real interest and the authors tell a compelling story. I would recommend the paper is accepted for publication, I have some very minor suggestions (including one I don’t think needs directly addressing by the authors) below.

Reviewer #3: This is a short report on a very interesting highly mutated SARS-CoV-2 variant found in a Missouri watershed.
It should be of substantial interest as it chronicles the extensive long-term evolution of this sample.

I only have a few minor comments:

Line 53: it is never introduced what "MO45" means.

***This has been clarified.***

The manuscript lacks a data availability statement on where the raw sequencing data can be found.

**This has been added. The raw sequences are all in SRA.**

Reviewer #2: • Dates on the X axis of Figure 1 have been cut off.

**Fixed.**

• One slight worry I have about the method used – specifically the Omicron exclusion primers – if, as the authors suggest, the cryptic lineages show convergent evolution with Omicron – isn’t there a concern that the sites covered by the primers could mutate in these cryptic lineages and result in a lack of sensitivity? For example site 368 and sites 371-373. I don’t think this is what is happening in this case (as the lineage is clearly still detected) but it might be worth considering outside the bounds of this study.

***We added this statement:***

***“It cannot be known for certain why the MO45 lineage was not detected again after October 2022.  We cannot rule out that the lineage simply became undetectable by our amplification scheme, though we routinely amplified different sections of the SARS-CoV-2 genome and have not detected any outlier sequences from this sewershed.”***
